# Supplementary material for: The epidemicity index of recurrent SARS-CoV-2 infections
Source: Nat Commun. 2021 May 12;12:2752. doi: 10.1038/s41467-021-22878-7 (PMC8115165; doi:10.1038/s41467-021-22878-7)
Supplement: Supplementary file 1 — Supplementary Information [file 41467_2021_22878_MOESM1_ESM.pdf]

# Supplementary Information to

# *The epidemicity index of recurrent SARS-CoV-2 infections*

Lorenzo Mari[1], Renato Casagrandi[1], Enrico Bertuzzo[2], Damiano Pasetto[2], Stefano Miccoli[3], Andrea Rinaldo[4,5], and Marino Gatto[1]

[1]Dipartimento di Elettronica, Informazione e Bioingegneria, Politecnico di Milano
[2]Dipartimento di Scienze Ambientali, Informatica e Statistica, Università Ca' Foscari Venezia
[3]Dipartimento di Meccanica, Politecnico di Milano
[4]Laboratory of Ecohydrology, École Polytechnique Fédérale de Lausanne
[5]Dipartimento ICEA, Università di Padova

## Supplementary Results

### Spatially heterogeneous transmission and preventive control measures

All the results reported in Figure 3 in the main text refer to a spatially homogeneous deployment of the containment measures, which may constitute a simple, yet reasonable preventive strategy, in particular if disease transmission is expected to occur homogeneously over the whole territory[1,2]. On the other hand, if transmission rates vary over space, a more targeted approach to containment could be that of focusing the control efforts in the most-at-risk areas, where the epidemic is likely to hit more severely.

An alternative parameterization of the model has been investigated to analyze the role possibly played by heterogeneous transmission in COVID-19 dynamics[1–3]. To that

end, the local values of the basic reproduction number estimated by Guzzetta *et al.*[4] have been used to compute region-specific values of the transmission rate $\beta^P$, namely by inverting the formula for local $R_0$ provided in the next section and using for all other parameter values those shown in Supplementary Table 1. Regions for which no estimates of the basic reproduction number are given in Guzzetta *et al.*[4] have been attributed the median value of the available regional figures therein. The values of $\beta_P$ at the scale of second-level administrative divisions have been then evaluated by assigning each spatial unit the value of the transmission rate of the region where that province or metropolitan city is located. Two different sets of estimates for the local $R_{0i}$ are provided in Guzzetta *et al.*[4], obtained from either hospital admission or symptom onset dates. Both sets have been retained for analysis and are given in Supplementary Table 1.

Some numerical results obtained by using these heterogeneous transmission rates and a spatially targeted approach are shown in Supplementary Figure 5. Specifically, in this example, provinces are split into two groups according to their local transmission rate. In higher-risk communities a stronger control (either transmission reduction or isolation of infected people, in this example) is then enforced. Trade-offs between spreading containment efforts over larger areas vs. focusing on smaller ones, or between exerting stronger control in the most-at-risk regions vs. opting for a more homogeneous approach do clearly emerge.

As an example, long-term disease transmission can be suppressed by a $\approx 70\%$ reduction of transmission applied uniformly at the country scale (see Supplementary Figure 4), as well as by a $\approx 90\%$ transmission reduction in 50% of the provinces (those where the highest transmission rates are estimated to occur) if the effectiveness of transmission reduction in the others is at least $\approx 55\%$, or by a $\approx 90\%$ transmission reduction in 10% of the provinces if the effectiveness of transmission reduction in the others (those

where the lowest transmission rates are projected) is at least $\approx 65\%$ (panel a of Supplementary Figure 5). In general, the curves identifying the conditions for long-term transmission suppression or positive epidemicity obtained with spatially heterogeneous controls depart rapidly from those obtained with homogeneous controls over wide ranges of parameter $\kappa$, which describes the relative containment effort in strongly vs. weakly controlled provinces.

Qualitatively similar trade-offs emerge when isolation of infected individuals is analyzed (panel b of Supplementary Figure 5). In this case, though, the curves identifying the conditions for long-term transmission suppression or positive epidemicity obtained with spatially heterogeneous controls run close to those obtained with homogeneous controls over a wide range of $\kappa$ values. This may suggest that a reasonable containment strategy could be that of enforcing transmission reduction, which can be achieved with relatively simple measures like social distancing, local mobility restriction, and the use of personal protective equipment, over large portions of the territory (possibly, the whole country), while at the same time focusing testing and isolation efforts (which by contrast require a complex sanitary organization) in the most-at-risk areas.

## Reactive control strategies

To evaluate the effects of control strategies that are deployed reactively once an epidemic outbreak has already started, epidemic trajectories need to be simulated by numerically integrating system (3) (Methods) text starting from a suitable initial condition. As the COVID-19 pandemic has made unfortunately evident, the early phase in the diffusion of an emergent pathogen may be cryptic, with the disease spreading undetected—and uncontrolled—in the population. Then, as soon as health authorities recognize the threat and start to react to it, containment measures begin to be deployed. The Italian lockdown

and similar experiences worldwide have shown that these measures need to be kept in place for weeks, if not months, before they can be cautiously released. In the following, we report on an extensive simulation exercise aimed to evaluate the role played by the location of the initial epidemic hotbed, as well as the type, spatial configuration, and strength of the containment measure(s) on the transmission dynamics of COVID-19, as described by SEPIAR applied to the Italian context.

We simulate a COVID-19 outbreak starting from one of the $n = 107$ Italian provinces or metropolitan cities (say $i$), initially with one exposed individual ($E_i(0) = 1$, $S_i(0) = N_i - 1$, all other variables are set to zero at the beginning of the experiment). Transmission dynamics are simulated numerically using the parameter values[2] of Table 2 (Methods) without any controls ($\epsilon_i = 0$ for all $i$'s, $\xi_{ij} = 0$ for all $i$'s and $j$'s, and $\chi_i^X = 0$ for all $i$'s and $X \in \{E, P, I, A\}$) until a pre-defined surveillance threshold is exceeded in one or more provinces (say the $k$th, possibly with $k \neq i$ because of the spatial structure of the model). Specifically, we use a threshold of 10 new symptomatic cases per 100 thousand population in one week. From that moment on, containment measures are deployed, focusing on the province(s) where the surveillance threshold has been crossed. The containment strategy is picked from a portfolio of different control types, which include i) transmission reduction: $\epsilon_k = \epsilon$ for all $k$'s, with $0 < \epsilon \leq 1$; ii) travel restriction, both incoming and outgoing: $\xi_{kj} = \xi_{jk} = \xi$ for all $k$'s and $j$'s, with $0 < \xi \leq 1$; iii) isolation of infected individuals: $\chi_k^X = \chi$ for all $k$'s and $X \in \{E, P, I, A\}$, with $\chi > 0$; iv) combined interventions: $\epsilon_k = \bar{\epsilon}$ for all $k$'s, $\xi_{kj} = \xi_{jk} = \bar{\xi}$ for all $k$'s and $j$'s, and $\chi_k^X = \chi$ for all $k$'s and $X \in \{E, P, I, A\}$, with $\chi > 0$; as well as spatial strategies (province(s) $k$ alone, $k$ and the ring(s) of nearest-neighbor provinces, the whole region(s) where province(s) $k$ is/are located, the whole region(s) plus the ring(s) of nearest-neighbor regions, the whole country. The simulation including controls is run for a timespan of six weeks,

starting from the deployment of the first containment measures. During this period of time, the surveillance threshold may be passed in other provinces, thus triggering the expansion of the area where controls are in place, according to the geographical rules outlined above.

To compare the effectiveness of different containment options and spatial configurations, diagnostic metrics like the total number of infections

$$c(t) = \sum_{i=1}^{n} \int_0^t \lambda_i(\zeta) S_i(\zeta) d\zeta \,, \tag{S1}$$

the average prevalence of infected individuals in the community (i.e excluding those who are quarantined or hospitalized)

$$p(t) = \frac{\sum_{i=1}^{n} E_i(t) + P_i(t) + I_i(t) + A_i(t)}{\sum_{i=1}^{n} S_i(t) + E_i(t) + P_i(t) + I_i(t) + A_i(t) + R_i(t)} \,, \tag{S2}$$

both evaluated at the country scale, and the effective reproduction number and epidemicity index, $\mathcal{R}_t$ and $e(t)$, are computed at the end of the simulation period (say, at $t = \tau$). The simulation is then repeated for different locations of the initial transmission focus, control types, spatial strategies, and intensities of the containment action.

Supplementary Figure 6 reports some results from the simulation exercise outlined above. All control types, except travel restrictions alone, can substantially reduce both the total count of cases (a–d) and the average prevalence of the infection (e–h) at the end of the simulation period. A clear gradient in effectiveness exists for different spatial strategies, with controls applied over wider domains (from single provinces to the whole country) progressively resulting in lower case count and infection prevalence. Indeed, only a country-wide approach can produce a sizable reduction of the effective reproduction number (i–l) and the effective epidemicity index (m–p), possibly below the threshold values $\mathcal{R}_t = 1$ and $e(t) = 0$, respectively. If met, the former condition determines the

suppression of long-term pathogen circulation, as demonstrated by the corresponding drop in disease prevalence; the latter guarantees against transient epidemic outbreaks, which might be triggered by the appearance of new transmission foci seeded e.g. by international travel. Like in the case of Figure 3 in the main text, the simultaneous deployment of multiple interventions seems to yield the most promising results in terms of control effectiveness, especially if operated at the country scale.

In addition to the baseline explored in Supplementary Figure 6, we have considered three other scenarios for the reactive implementation of containment measures. First, we have modified the surveillance threshold whose crossing triggers the deployment of control actions. Specifically, in Supplementary Figure 7 we have used a value of 10 new infections (not necessarily symptomatic) per 100 thousand population in one week. Clearly, this threshold is more stringent than the previous one, as not all exposed individuals would eventually develop symptoms. It also allows a more responsive deployment of control measures, as symptoms may take days to become manifest. Together, these features may lead to an earlier containment of disease transmission, which in turn results in lower case count and infection prevalence (but no observable differences on the effective reproduction number and epidemicity index). On the other hand, it must be noted that a surveillance threshold based on exposed rather than symptomatic individuals is also more difficult to implement, because it requires an efficient monitoring system able to recognize infected people at an early stage of their clinical course, as well as asymptomatic and paucisymptomatic carriers. Second, we have considered an alternative parameterization of the model that makes use of regional estimates[4] of the basic reproduction number in the early phase of the Italian COVID-19 epidemic (Supplementary Table 1, $R_0^{\mathrm{hosp}}$). The results, shown in Supplementary Figure 8, are both qualitatively and quantitatively consistent with those obtained with the baseline

scenario of homogeneous transmission parameters (Supplementary Figure 6), despite small differences in the estimate of the effective reproduction number. Third, we have modified the initial condition to allow for multiple transmission foci to be present at the beginning of the epidemic. In this case, the 10 provinces where the outbreak starts (with one exposed individual each) are selected randomly, with the random extraction procedure being replicated $10,000$ times. Despite a $\approx 60\%$ increase in the number of cases and disease prevalence in the absence of controls (a byproduct of the change in the initial condition), the results shown in Supplementary Figure 9 are once again both qualitatively and quantitatively consistent with those of the baseline scenario.

## References


1. Gatto, M. *et al.* Spread and dynamics of the COVID-19 epidemic in Italy: Effects of emergency containment measures. *Proceedings of the National Academy of Sciences USA* **117,** 10484–10491. doi:10.1073/pnas.2004978117 (Apr. 2020).
2. Bertuzzo, E. *et al.* The geography of COVID-19 spread in Italy and implications for the relaxation of confinement measures. *Nature Communications* **11,** 4264. doi:10.1038/s41467-020-18050-2 (Aug. 2020).
3. Li, R. *et al.* Substantial undocumented infection facilitates the rapid dissemination of novel coronavirus (SARS-CoV2). *Science* **368,** 489–493. doi:10.1126/science.abb3221 (Mar. 2020).
4. Guzzetta, G. *et al.* The impact of a nation-wide lockdown on COVID-19 transmissibility in Italy. *arXiv.* arXiv: 2004.12338 [q-bio.PE]. https://arxiv.org/abs/2004.12338 (2020).

# Supplementary Figures

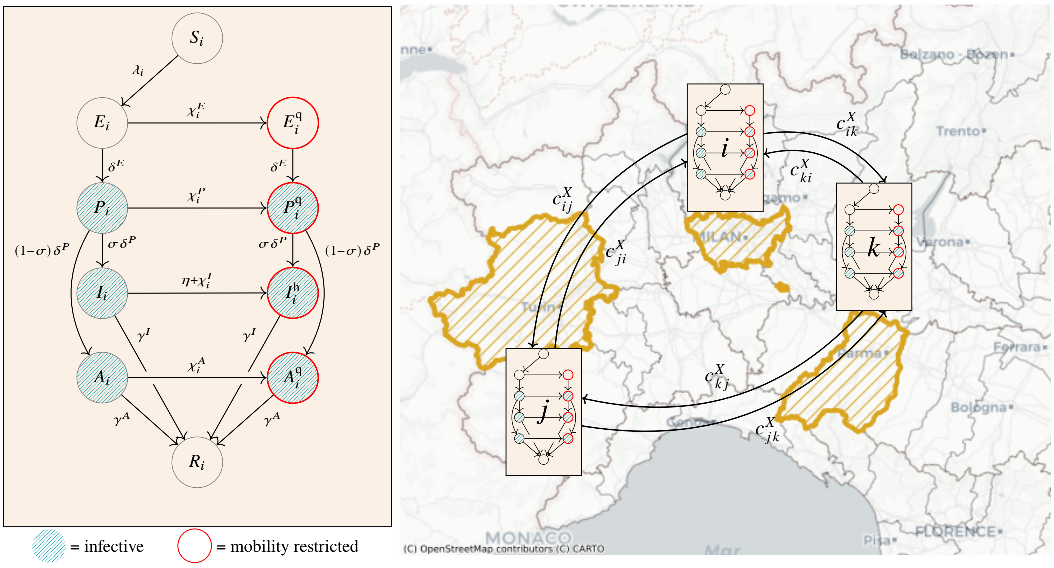


**Supplementary Figure 1: Schematic representation of the local transmission dynamics and the spatial structure of the model**. (Left panel) Local dynamics of the SEPIAR model, as in Eqs. (3) (Methods); (Right) Sketch of the connections among three local communities ($i$, $j$, $k$) out of a total $n = 107$ nodes at the second administrative level (provinces and metropolitan cities).

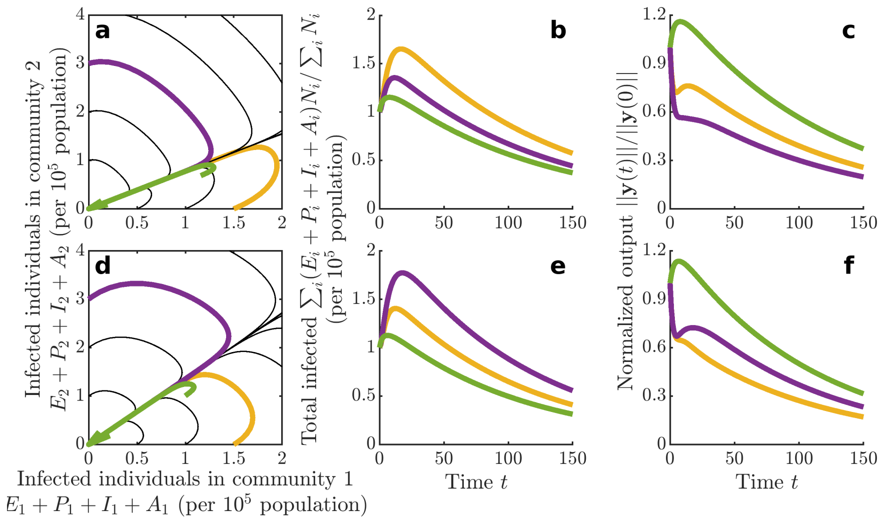


**Supplementary Figure 2: Further examples of the concept of epidemicity**. Details and parameters as in Figure 1 in the main text, except for (a–c) $\beta_1^P = 5.2 \cdot 10^{-1}$ days$^{-1}$, $\beta_2^P = 7.0 \cdot 10^{-2}$ days$^{-1}$ (point ④ in Figure 2 in the main text); (d–f) $\beta_1^P = 3.4 \cdot 10^{-1}$ days$^{-1}$, $\beta_2^P = 8.4 \cdot 10^{-1}$ days$^{-1}$ (point ⑤ in Figure 2 in the main text). For both parameter combinations, $\mathcal{R}_0 < 1$ and $e_0 > 0$. As in panels d–f of Figure 1 in the main text, all trajectories converge to the DFE (a and d), but disease prevalence has a peak, before declining over time (b and e). Also, for suitable initial conditions, a transitory increase of the system output following a pulse perturbation is possible (c and f).

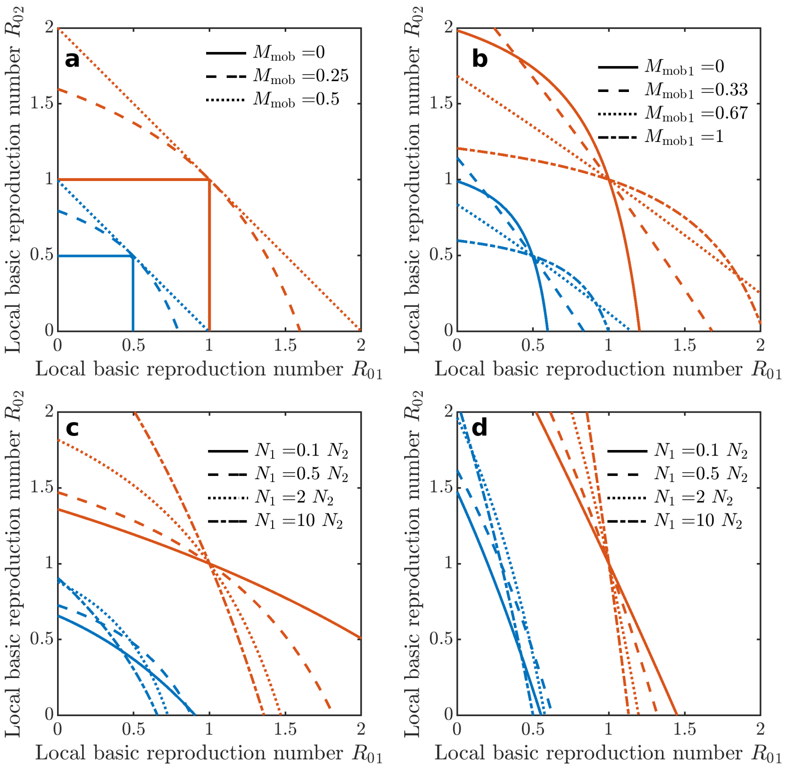


**Supplementary Figure 3: The role of population distribution and human mobility in determining the long- and short-term behavior of the two-patch SEPIAR.** The red curves identify the condition $\mathcal{R}_0 = 1$, whereas the blue curves identify $e_0 = 0$. Endemic transmission is possible above the red curves ($\mathcal{R}_0 > 1$), while short-term epidemicity may be present above the blue curves ($e_0 > 0$). (a) $N_1 = N_2$, $M_{12}^{S,E,P,A} = M_{21}^{S,E,P,A} = M_{\mathrm{mob}}$. (b) $N_1 = N_2$, $M_{12}^{S,E,P,A} = M_{\mathrm{mob}1}$, $M_{21}^{S,E,P,A} = 0.5$. (c) $M_{12}^{S,E,P,A} = M_{21}^{S,E,P,A} = 0.25$. (d) $M_{12}^{S,E,P,A} = 1/10$, $M_{21}^{S,E,P,A} = 2/3$. In all cases, $M_{12}^{I} = M_{21}^{I} = 0$. Other parameters and details as in Figures 1 and 2 in the main text.

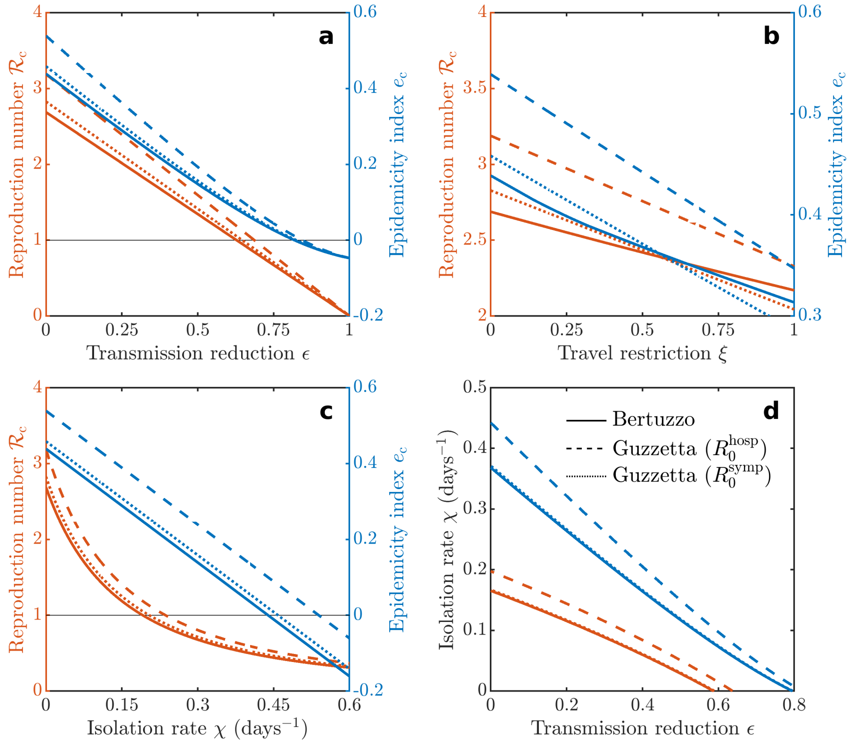


**Supplementary Figure 4: The effects of spatially homogeneous control measures on $\mathcal{R}_c$ and $e_c$ of COVID-19 in Italy with heterogeneous transmission rates.** Different line styles show the result of different assumptions concerning the spatial homogeneity (displayed for comparison) or heterogeneity of the transmission parameters. (a) Plot of the relation between $\mathcal{R}_c$ (red curves, left axis), $e_c$ (blue curves, right axis) and the transmission rate reduction ($\epsilon_i = \epsilon$ for all $i$'s); (b) same as (a) for the effects of travel restrictions ($\xi_{ij} = \xi$ for all $i$'s and $j$'s); (c) same as (a) for the effects of the isolation of infected individuals ($\chi_i^X = \chi$ for all $i$'s and $X \in \{E, P, I, A\}$); (d) Simultaneous deployment of controls leading to $\mathcal{R}_c = 1$ or $e_c = 0$ for $\xi$, the imposed travel restriction, equal to 0.5. The DFE is unstable below the red curves ($\mathcal{R}_c > 1$), while short-term epidemics are not possible above the blue curves ($e_c < 0$). In all panels, $w^X = 1$ ($X \in \{E, P, I, A\}$). Homogeneous transmission has been parameterized according to Bertuzzo *et al.*[2] (Table 2), while a study by Guzzetta *et al.*[4] has been used to characterize heterogeneous transmission (Supplementary Table 1).

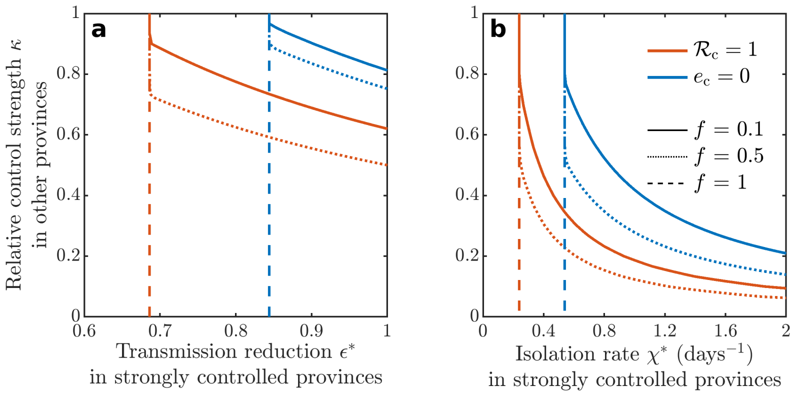


**Supplementary Figure 5: Spatially heterogeneous measures for the preventive containment of COVID-19.** Controls (a, transmission reduction; b, isolation of infected individuals) are more strongly enforced in a fraction $0 \leq f \leq 1$ of provinces, where $\epsilon_i = \epsilon^*$ (a) or $\chi_i^X = \chi^*$ (b), than in the others, where instead $\epsilon_i = \kappa\epsilon^*$ (a) or $\chi_i^X = \kappa\chi^*$ (b), with $X \in \{E, P, I, A\}$ and $\kappa \leq 1$. The deployment of controls is prioritized according to the local values of the transmission rate evaluated via the estimates of the regional basic reproduction number ($R_0^{\text{hosp}}$) provided in Guzzetta *et al.*[4] (Supplementary Table 1). Long-term transmission and short-term epidemicity are shut down on the right of the red ($\mathcal{R}_c = 1$) or blue ($e_c = 0$) curves, respectively. The effect of travel restrictions $\xi_{ij}$ is not shown because this family of containment measures allows suppression of neither long-term transmission nor short-term epidemicity, at least for the considered parameter values (Table 2; $w^X = 1$, with $X \in \{E, P, I, A\}$).

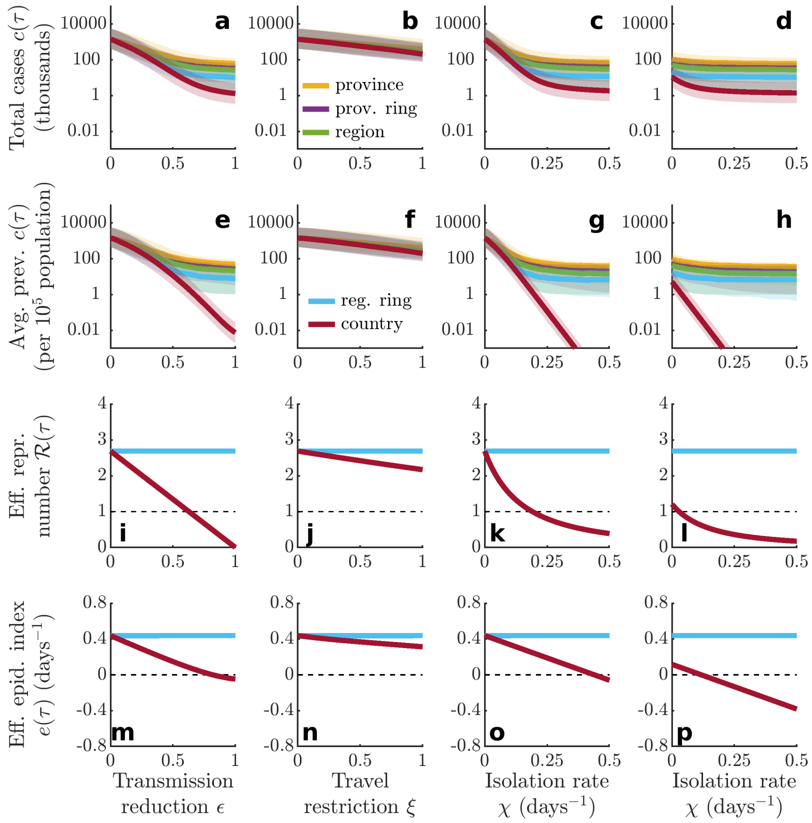


**Supplementary Figure 6: Spatially heterogeneous measures deployed reactively in the course of a COVID-19 epidemic.** Model (3) (Methods) is simulated starting from the disease-free equilibrium perturbed by one exposed individual in one province. The simulation proceeds in the absence of controls until a surveillance threshold (10 new symptomatic infections per 100 thousand population in a week) is exceeded in at least one province. Then, containment measures are deployed, and the simulation is run for other six weeks, after which some epidemiological indicators are evaluated to compare different control measures and spatial strategies (Supplementary Results). (a–d) Cumulative number of country-wide infections (a: transmission reduction; b: travel restriction; c: isolation of infected individuals; d: simultaneous deployment of all controls, with $\bar{\epsilon} = \bar{\xi} = 0.5$ in the target provinces). (e–h) Average country-wide prevalence of infection. (i–l) Effective reproduction number. (m–p) Effective epidemicity index. In each panel, colors code different spatial strategies (controls deployed to target provinces only, or also to: a ring of nearest-neighbor provinces, the whole administrative region, a ring of nearest-neighbor regions, or the whole country). The lines and the shaded areas indicate the median and the 90% percentile range, respectively, of the epidemiological indicators evaluated for different initial conditions, corresponding to seeding the epidemic in each of the $n = 107$ Italian provinces, one at a time. Parameters as in Table 2, with $w^X = 1$ ($X \in \{E, P, I, A\}$).

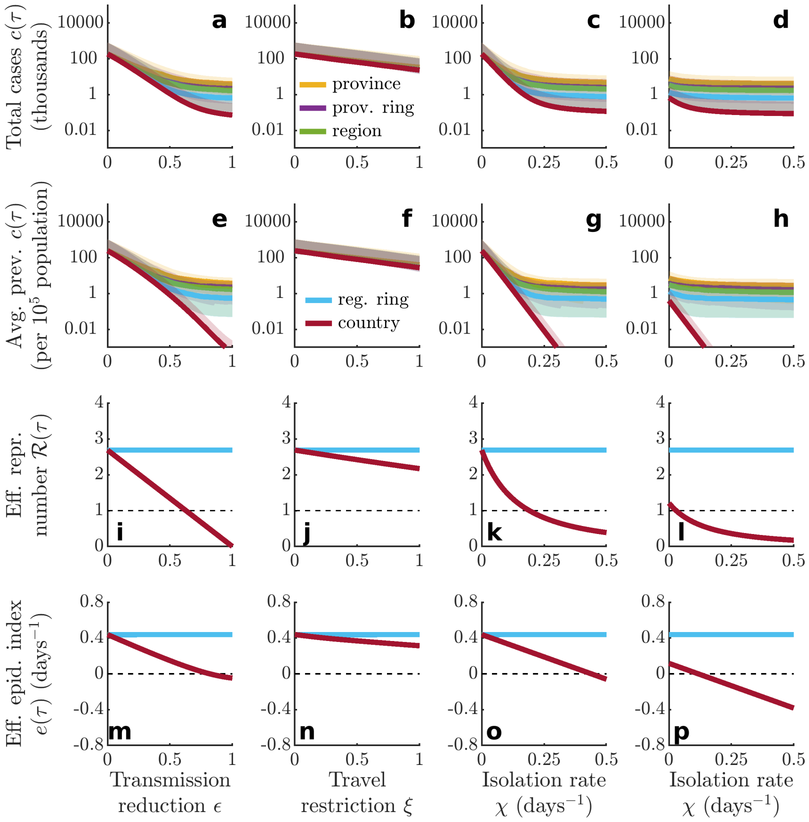


**Supplementary Figure 7: Spatially heterogeneous measures deployed reactively in the course of a COVID-19 epidemic (alternative surveillance threshold).** Details as in Supplementary Figure 6, with a more stringent surveillance threshold (10 new cases, be they symptomatic or not, per 100 thousand population in one week). See Supplementary Results for other technical details.

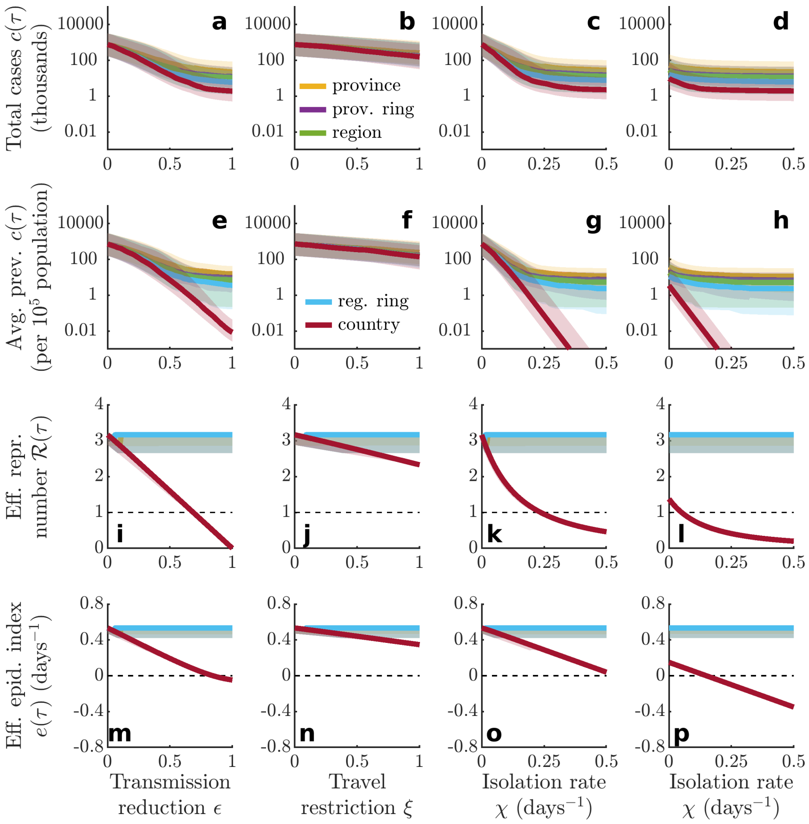


**Supplementary Figure 8: Spatially heterogeneous measures deployed reactively in the course of a COVID-19 epidemic (alternative model parameterization).** Details as in Supplementary Figure 6, with spatially heterogeneous values of the transmission parameters (Supplementary Table 1, $R_0^{\text{hosp}}$). See Supplementary Results for other technical details.

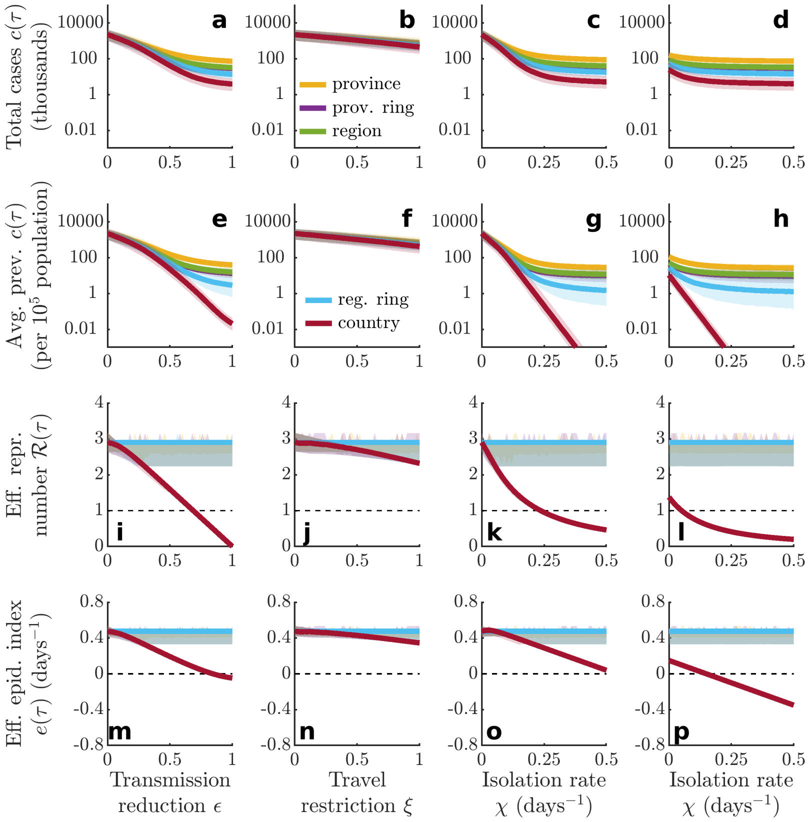


**Supplementary Figure 9: Spatially heterogeneous measures deployed reactively in the course of a COVID-19 epidemic (alternative initial conditions).** Details as in Supplementary Figure 6, with 10 randomly selected initial transmission foci. In this case, the lines and the shaded areas indicate the median and the 90% percentile range, respectively, of the epidemiological indicators evaluated over $10,000$ replicas of the random extraction process. See Supplementary Results for other technical details.

# Supplementary Tables

| **Region** | $R_0^{\text{hosp}}$ | $R_0^{\text{symp}}$ | $\beta^{P,\text{hosp}}$ (days$^{-1}$) | $\beta^{P,\text{symp}}$ (days$^{-1}$) |
|---|---|---|---|---|
| Lombardy | 1.68 | 1.48 | $7.87 \cdot 10^{-1}$ | $6.92 \cdot 10^{-1}$ |
| Bolzano | 2.31 | 2.84 | 1.08 | 1.33 |
| Trento | 3.23 | 2.44 | 1.51 | 1.14 |
| Veneto | 2.01 | 2.07 | $9.45 \cdot 10^{-1}$ | $9.69 \cdot 10^{-1}$ |
| Friuli-Venezia Giulia | 2.24 | 2.18 | 1.05 | 1.02 |
| Emilia-Romagna | 1.54 | 1.58 | $7.20 \cdot 10^{-1}$ | $7.41 \cdot 10^{-1}$ |
| Tuscany | 2.64 | 2.05 | 1.24 | $9.60 \cdot 10^{-1}$ |
| Lazio | 1.79 | 2.40 | $8.41 \cdot 10^{-1}$ | 1.13 |
| Apulia | 2.66 | 2.03 | 1.25 | $9.51 \cdot 10^{-1}$ |
| Sardinia | 2.90 | 2.16 | 1.36 | 1.01 |
| Median | 2.24 | 2.06 | 1.05 | $9.69 \cdot 10^{-1}$ |

**Supplementary Table 1:** Regional estimates of the basic reproduction number at the beginning of the COVID-19 epidemic in Italy according to Fig. 1b in Guzzetta et al. (2020)[4]. The two different regional values of the basic reproduction number refer to estimates obtained by using either hospital admission ($R_0^{\text{hosp}}$) or symptom onset ($R_0^{\text{symp}}$) dates. The local values of $\beta^P$ have been obtained from the formula of the basic reproduction number reported in the main text (Eq. (1)). Regions for which no estimates of the local reproduction number were available from[4] have been attributed the median values reported in the last row of the table.
